# Getting ON-TRAC, a team-centred design study of a reflexivity aid to support resuscitation teams’ information sharing

**DOI:** 10.1186/s41077-025-00340-8

**Published:** 2025-03-28

**Authors:** Lars Mommers, Dennie Wulterkens, Steven Winkel, Bas van den Bogaard, Walter J. Eppich, Walther N. K. A. van Mook

**Affiliations:** 1 Department of Simulation in Healthcare, MUMC, Maastricht, the Netherlands; 2 Department of Anaesthesiology and Pain Medicine, MUMC, Maastricht, the Netherlands; 3 Medical Quality Training Institute, Nieuwegein, The Netherlands; 4 Department of Intensive Care Medicine, OLVG, Amsterdam, The Netherlands; 5 Faculty of Medicine, Dentistry and Health Sciences, University of Melbourne, Melbourne, Australia; 6 School of Health Professions Education, Maastricht University, Maastricht, The Netherlands; 7 Academy for Postgraduate Medical Training, MUMC, Maastricht, The Netherlands; 8 Department of Intensive Care Medicine, MUMC, Maastricht, The Netherlands

**Keywords:** Team reflexivity, Team reflection, Non-technical skills, Team performance, Team mental model, Shared mental model, Cognitive aid, Simulation, Resuscitation

## Abstract

**Background:**

Effective information sharing is crucial for emergency care teams to maintain an accurate shared mental model. This study describes the design, simulation-based testing and implementation of a team reflexivity aid to facilitate in-action information sharing during resuscitations.

**Methods:**

A five-phase team-centred iterative design process was employed. Phase 1 involved a literature review to identify in-action cognitive aids. Phase 2 focused on conceptual design, followed by simulation-based testing and modifications in phase 3. Implementation through simulation-based user training occurred in phase 4 at a large non-university teaching hospital. Phase 5 evaluated the aid among resuscitation team members in the emergency department after one year.

**Results:**

The *phase 1* literature review identified 58 cognitive aids, with only 10 designed as ‘team aid’. Studies using team information screens found increase team and task performance in simulation-based environments, with no evaluations in authentic workplaces. *Phase 2* resulted in a three-section team reflexivity aid, iteratively modified in three rounds of simulation-based testing (*N* = 30 groups) *phase 3* resulted in a team reflexivity aid containing five sections: resuscitation times and intervals, patient history, interventions on a longitudinal timeline, differential diagnosis and a quick review section. *Phase 4* consisted of reflexivity aid user training with simulation-based education (*N* = 60 sessions) and the creation of a digital entry form to store data in the patient’s electronic medical record. Evaluation after one year in *phase 5*, (*N* = 84) showed perceived improvements in communication (3.82 ± 0.77), documentation (4.25 ± 0.66), cognitive load (3.94 ± 0.68), and team performance (3.80 ± 0.76) on a 5-point Likert scale. Thematic analysis of user feedback identified improvements in both teamwork and taskwork. Teamwork enhancements included better situation awareness, communication and team participation. Taskwork improvements were seen in drug administration and clinical reasoning.

**Conclusions:**

This study demonstrated the successful development and implementation of a Team Reflexivity Aid for Cardiac arrests using simulation methodology. This task-focused team tool improved perceived team situation awareness, communication, and overall performance. The research highlights the interplay between task- and teamwork in healthcare settings, underscoring the potential for taskwork-oriented tools to benefit team dynamics. These findings warrant further investigation into team-supportive interventions and their impact on resuscitation outcomes.

**Supplementary Information:**

The online version contains supplementary material available at 10.1186/s41077-025-00340-8.

## Background

Information sharing and processing are critical processes for emergency care teams in order to construct and maintain an accurate shared team mental model necessary for optimal team performance and good patient outcomes [1–4]. However, these processes are under constant stress as such teams respond on an ad-hoc basis [4, 5], while managing an overload of (incomplete) information [6–8] and performing concomitant life-saving tasks or dealing with distractions from other patients or noisy emergency departments [9]. Team leaders are encouraged to think out loud and query other team members for cross-checks, observations and feedback on the team’s performance [4]. The stressful and chaotic working conditions in which this is to occur [10] can however negatively impact individual performance by delaying memory retrieval [11], shifting communication towards implicit coordination [12], predisposing task fixation [13], reducing attentional resources [14] and impairing overall performance [15, 16]. Cognitive aids are often suggested to improve performance in emergency situations [17, 18]. Traditional aids often provide simplified ‘decision support’ steps, guiding the team (leader) on ‘what to do’. In contrast, this study focuses on a cardiac arrest reflexivity aid facilitating information sharing within resuscitation teams, leaving the decision-making to the team’s cognition.

A ‘team’ is defined as ‘two or more people who have defined roles and depend on each other to accomplish a shared goal’ [19]. To achieve their shared goal of effective patient care, the team needs sufficient *taskwork*, i.e. guideline-derived interventions, as well as sufficient *teamwork*. The latter helps to direct, align and monitor the taskwork [20]. Examples of teamwork skills include closed-loop communication, mutual trust and a common understanding of the team’s resources, e.g. members’ knowledge, skills and experiences, goals and objectives under which the team operates [12]. This common psychological understanding is often referred to as a team’s shared mental model (SMM). Actions resulting from an accurate SMM include the provision of guidance, support or assistance *before* being asked to assist, and regularly sharing updates on the team’s situation awareness [21]. An accurate SMM is associated with more effective communication, more intrinsic performance monitoring [22], increased team backup behaviour, better adaptability and improved performance compared to teams lacking an accurate SMM [7, 12, 21, 23–25]. To construct, update, and maintain this SMM, teams need to communicate and share their insights to align all individual perspectives and perceptions into a team’s cognition. The theoretical concept of this ‘reflection’ process can be traced back to Donald Schön’s reflective practitioner [26]. Michael West [27, 28] expanded this individual practitioner’s stance into the concept of ‘team reflexivity’ (TR). TR is defined as ‘the extent to which group members overtly reflect upon and communicate about the group’s objectives, strategies and processes and adapt them to current or anticipated circumstances’ [29]. This concept extends beyond simple reflection, incorporating adaptive planning that distinguishes it from mere ‘team reflection’ [30]. At its core, TR can be viewed as a team communication process requiring collective reflection [31]. This communicative process can be subdivided into initiation of information sharing, co-construction of information and constructive resolution of different interpretations (i.e. conflicts) [32]. Schmutz et al. [29, 31] further conceptualised TR in healthcare, delineating specific team reflexive behaviours that occur during patient care episodes.

When TR occurs in parallel with ongoing patient care, this is referred to as ‘in-action’ TR. Empiric work has shown that especially larger teams, improve their team performance whenever they engage in in-action TR [31]. However, ongoing patient care poses tremendous challenges for emergency care teams as context, information, and goals are dynamically evolving, not all team members are always available for TR processes, and time constraints apply [31]. Thus, cognitive aids have been proposed to facilitate external initiation and structuring of team processes such as in-action TR [15, 16, 25, 33].

### Cognitive aids

Cognitive aids are well known for their application in emergencies. The broader term ‘cognitive aid’ however encompasses a myriad of applications, including checklists, alarms, physical tools, mobile applications (apps), mnemonics and reminders [34]. In healthcare, cognitive aids often refer to any tool that that helps professionals remember, determine, or act upon key information [35, 36].

Properly designed cognitive aids in healthcare have been found to reduce omissions [18, 37–39], improve guideline adherence [18, 36–38, 40] and enhance attention distribution [41]. They can also decrease mental workload [42], improve non-technical skills [43, 44], increase efficacy [38, 45] and enhance overall resuscitation team performance [37, 42, 45]. However, cognitive aids might also have undesirable effects, such as increased workload [46, 47], reduced efficacy [39, 48–50], inattentional blindness and fixation error [39, 41, 51] and distractions [52, 53]. They can also lead to persistent errors [51, 54, 55], fear of appearing unprofessional [56] or inadvertent selection of the wrong aid [51], which can result in overall reduced performance [57, 58]. Furthermore, cognitive aids not only affect individual cognition, but rather influence the dynamics within the entire team [59]. Proper design, implementation and evaluation of cognitive aids in the appropriate context are therefore crucial yet often overlooked [7, 34, 39, 58, 60].

In summary, information sharing and processing are crucial for emergency care teams to establish and maintain an accurate SMM needed for optimal team performance. Given the dynamic and stressful conditions under which these teams operate, it is important to explore the extent to which a ‘team reflexivity aid’ can support important information sharing and processing. A specific example of such an emergency care team is a hospital’s resuscitation (code) team. This paper describes the design, simulation-based testing and implementation of a team reflexivity aid to support information sharing amongst resuscitation teams.

## Objective

To develop a team aid using an iterative user-centred design process with the objective to support resuscitation teams’ in-action reflexivity.

## Materials and methods

### Study design

**Fig. 1:**
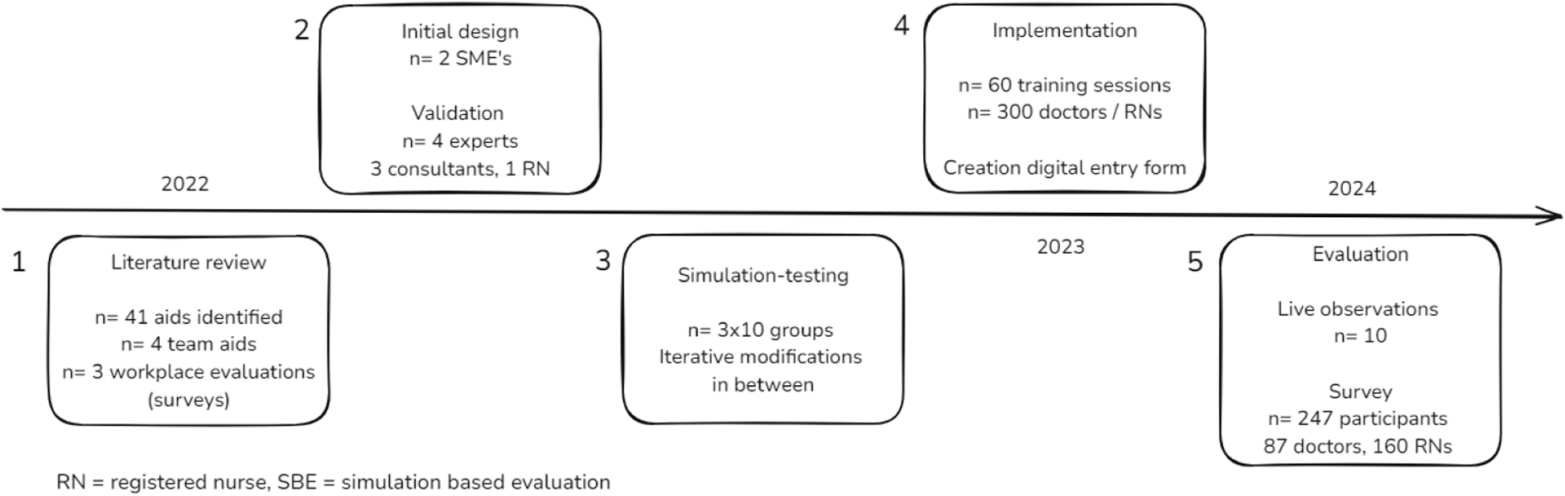
Overview of study design and results

We conducted a five-phase, team-centred iterative design process consisting of a literature review, conceptual design of the team reflexivity aid, iterative simulation-based testing and re-design, implementation and authentic workplace evaluation in the emergency department after one year [61]. Figure 1 schematically illustrates the consecutive phases of the process. We apply the reporting guideline on healthcare simulation research and STROBE extension wherever applicable [62, 63].

### Ethics

According to the hospital’s Institutional Review Board, this study did not involve Medical Research Involving Human Subjects (WMO) and was part of routine educational activities and was therefore exempt from full ethics review. The study adhered to ethical principles for educational research, no identifiable participant data was collected, and all data generated through this project was stored on secure password-protected devices and servers. Participation was entirely voluntary throughout all phases. Individuals provided informed consent and could discontinue their involvement at any point during the study.

### Literature review (phase 1)

A literature review was conducted in MEDLINE, EMBASE and Cochrane up until May 2022 and updated before publication. The search string included “heart arrest” [MESH] and “resuscitation” combined with “team aid”, “team tool” or “cognitive aid”. Relevant articles’ bibliographies were additionally hand-searched. Titles and abstracts were screened by two researchers (LM, DW). Inclusion was set on studies describing cognitive aids in the broadest sense designed to be used during ‘in-action’ resuscitation care. Studies on devices exclusively available in training sessions (e.g. connected mannikin feedback devices) or innovative technologies to ‘call for assistance’ (e.g. Google Glass or telemedicine) were excluded.

### Conceptual design (phase 2)

Two subject-matter experts (SMEs) developed the conceptual design of the team aid based on the literature review. The first SME (LM) is a consultant anaesthesiologist, helicopter emergency physician and advanced life support instructor with 15 years of experience in (pre)hospital emergency medical care. The second SME (DW) is a registered nurse with over 40 years of international experience in (pre)hospital emergency medical care and over 15 years of experience in postgraduate crew resource management training.

A multidisciplinary panel consisting of three consultants—in emergency medicine, cardiology and intensive care medicine—and one registered nurse, all experienced advanced life support instructors with at least 15 years of clinical experience, provided their feedback on design and content.

### Simulation-based testing (phase 3)

Iterative rounds of testing and modifications of the team aid were conducted during regular advanced life support simulation training sessions. These sessions were part of the ongoing re-certification course for resuscitation team members. Participants were conveniently sampled from various hospitals with a range of small to large-volume resuscitation centres. Our sample for this phase included doctors and nurses. Each training session lasted one day and included three groups of five to six participants. Each group was presented with six ‘immersive’ (sometimes referred to as ‘high-fidelity’) simulation scenarios: witnessed ventricular fibrillation, refractory ventricular fibrillation, pulmonary embolism, hypertrophic cardiomyopathy, subarachnoid haemorrhage and electrolyte disorder. The equipment used was a Laerdal Resusci Anne QCPR mannikin with Advanced Airway Head (Laerdal Medical, Stavanger, Norway) on an adjustable patient gurney, a REALITi 360 advanced patient monitor and defibrillator (iSimulate, Albany, NY, USA), stocked code cart and two 55-inch television screens. One screen mirrored the patient monitor, the other was used to display optional resources, e.g. ECGs, blood gas analysis results, ultrasound clips, X-ray images. Each scenario started with a short case vignette, followed by a pre-action preparation time of 2–3 min. Thereafter the team was given a short handover and the scenario started. The total scenario duration was 10 to 15 min, followed by a learner-centred facilitated debrief [64]. Debriefs lasted 30–40 min and were facilitated by two experienced clinicians and simulation experts using the PEARLS approach [65].

In terms of the team reflexivity aid, each group received a single-use, A_1_-sized paper-based version of the team aid well before the final simulation scenario, allowing participants time to familiarise themselves with the team composition and the simulation environment before using the tool for the first time. All participants’ feedback was written on the paper aids themselves, which were all collected afterwards for subsequent analysis. Feedback was analysed in iterative rounds by two researchers (LM, DW) and corresponding modifications to the team reflexivity aid were made until no further improvements were identified.

### Implementation and training (phase 4)

The implementation was conducted in a large Dutch non-university teaching hospital. The hospital includes cardiothoracic and interventional cardiology, an Extracorporeal-CardioPulmonary Resuscitation (E-CPR) program and admits approximately 125 OHCA patients per year.

Training in the use of the team aid was incorporated into the hospital’s resuscitation training program. Mandatory 2-h sessions were delivered once a week for groups of 5–10 healthcare professionals (doctors and nurses) from the hospital’s resuscitation team. Each resuscitation team member was scheduled to be (re)trained every six months. Each training session had a similar composition: (a) basic life support skill training, (b) a rapid-cycle deliberate practice for rhythm checks skill training, (c) a short plenary introduction of the team reflexivity aid for 15 min. Thereafter, a minimum of four and a maximum of five scripted resuscitation scenarios were conducted (depending on available time). The equipment used was an Ambu Man Advanced CPR mannikin (Ballerup, Denmark: Ambu A/S) on an adjustable patient gurney, a REALITi 360 advanced patient monitor and defibrillator (iSimulate, Albany, NY, USA) and stocked code cart as used in the hospital. Scenarios included myocardial infarction, pulmonary embolism, hypertrophic cardiomyopathy and intoxication. Each scenario had dedicated learning goals, including demonstration of safe (synchronised) defibrillation, correct adaption and switching between shock-/no-shock-rhythms and effective teamwork including (closed-loop) communication, effective leader-/followership and optimal task distribution. During each scenario, participants had the team reflexivity aid available. At the end of each scenario, a 10–15 min instructor-centred debrief was conducted using the Plus-Delta approach [66]. Particular focus was given to the team reflexivity aid, thereby providing a deliberate practice of five consecutive scenarios with the new team tool.

To facilitate implementation, a similar digital input form of the team reflexivity aid was created in the hospital’s patient management system for data entry and storage after each authentic resuscitation in the clinical environment.

### Evaluation in practice (phase 5)

The team reflexivity aid was evaluated through live observations and an online survey.

#### Observations

From January to June 2024, the hospital’s resuscitation coordinator who was not part of the resuscitation team observed all emergency department resuscitation team deployments for (code blue) OHCA resuscitations or in-situ process evaluations through simulation cases during regular working hours. Multidisciplinary resuscitation teams consisted of a consultant in intensive care medicine (team leader), one consultant in emergency medicine and three registered nurses from the emergency and/or coronary care department. Observers took field notes on task work aspects, e.g. correctness of medication, teamwork aspects, e.g. communication and user aspects of the team reflexivity aid, e.g. attention of team members to the tool.

#### Online survey

An online evaluative survey was created using Qualtrics™ to assess team members’ perceived usability of the new tool. The survey consisted of four sections: demographic data, layout data, content data and team effects. It was pilot-tested among colleagues (*N* = 5) not participating in the final evaluation, leading to two minor textual changes and the addition of a ‘back button’ to review and modify answers. The final survey included 17 questions: 8 multiple choice, 15 Likert-scale (5 items) and 3 open questions. The number of questions varied based on responses due to Qualtrics’ adaptive questioning feature.

All departments involved in the resuscitation team received an email invitation to the survey. Participation was voluntary, with no collection of personally identifiable or traceable data with all questions optional to answer. The survey was open for responses from January to March 2024, with bi-weekly reminders to maximise response rates. Participants had one week to complete the survey, after which the system would close their record and mark their attempt as incomplete. Partially completed surveys were included in the analysis to limit non-response bias. Raw data were stored on the Qualtrics platform (Qualtrics™, Provo, UT, USA). Respondents were prevented from taking the survey more than once using Qualtrics’ cookie-based functionality.

### Bias

We minimized confirmation bias in the design phase by starting with a literature review, including two subject matter experts on resuscitation and team performance with different backgrounds. We validated the content of the team aid with a multidisciplinary panel of experts from different hospitals and backgrounds. We also considered potential selection bias during phase three by evaluating the aid within the recertification course, thereby eliminating an individual selection process. This inevitably resulted in a selection bias of previously trained individuals, although this was the designated target group to use the team reflexivity aid. Neither of the experts involved in phases one or two were working in the hospital where the tool was introduced (phases 4 and 5), thereby limiting affinity bias. To limit observer bias and positivity bias with survey respondents, we purposefully combined the survey and the observations for the evaluation phase. Two-weekly reminders were sent to limit non-response bias amongst the survey participants.

### Statistical analysis

Quantitative data were analysed using IBM SPSS Statistics for Windows, Version 28.0.0.0 (IBM Corp.©). Multiple-choice and Likert-scale questions were analysed using descriptive statistics and reported as mean and standard deviation (SD) or count (N) and percentages (%) where applicable.

Qualitative data (field notes, observational data and open survey questions) were analysed using thematic analysis [67] when data richness was deemed sufficient for coding by two researchers (LM, SW). Analysis was conducted through: data familiarisation, initial coding, theme development, theme review and refinement, theme definition and naming, and reporting [67]. During coding and theme construction, any discrepancies were discussed until a consensus was reached.

## Results

An overview of the different study phases and participant numbers is schematically displayed in Fig. 1.

### Literature review (phase 1)

A literature review on cognitive aid application *during* cardiopulmonary resuscitation identified 58 aids (see Supplementary material 1). Most aids were designed for individual users, although ten aids were identified and designed for entire resuscitation teams [9, 52, 68–78]. These team-focused studies utilised large (video) screens, incorporating decision support steps [52, 68, 69, 71, 77], patient information [9, 68, 75] and/or documented interventions [9, 68, 69, 75] or drug preparation instruction [78]. Three interventions designed to improve team collaboration were identified: numbered jerseys [76] and two predefined role and task allocation systems—the A-C-L-S teamwork model [72] and CARD system [70, 73].

Studies analysing ‘*team screens*’ found beneficial effects on protocol adherence [52, 69], participants’ safety feeling [52] and team performance [9, 75, 79, 80]. However, they did not consistently report improvements in teams’ situation awareness [75] with some even reporting a decreased situation awareness due to fixation error and distraction [9, 52]. Most of the studies were conducted in simulation environments, highlighting the importance of simulation-based testing for new team tools. Of particular interest was the simulation study by Watkins, de Oliveira Filho, Furse, Muffly, Ramamurthi, Redding, et al. 2022 [81] comparing three different team aids: a technical, non-technical and combined decision-support tool (DST) for cardiac arrest. Technical performance increased when either the technical or combined DST was used and deteriorated when the non-technical DST was used; non-technical performance on the other hand was identical with either DST and comparable to team performance without a DST [81].

Four studies were conducted in authentic workplaces [35, 72, 82, 83]. Three of these evaluated the use of an emergency manual [35, 82] or code card [83] through surveys. Chong, Chou, Chiang, Wang, Liu, Ko, et al. 2024 [72] analysed the effects of their A-C-L-S team framework through video observations in the emergency department, finding an improved task performance over a 2-year period.

The design of the new team reflexivity tool was scaffolded by key principles derived from human factors and ergonomics research and cognitive aid design recommendations [84–91]. Flexibility and adaptability were prioritised to accommodate diverse note-taking styles and maximise applicability across various resuscitation scenarios, including both adult and paediatric cases [92]. Efficiency was emphasised through the implementation of rapid annotation capabilities with intuitive user guidance, and a clear structure allowing navigation between components [93]. Principles of familiarity and validity were addressed by incorporating relevant terminology and content derived from scientific literature, ensuring alignment with established resuscitation protocols [34, 61]. Physical ergonomics considerations were applied to accommodate handwriting and ensure appropriate sizing for readability and accessibility to all team members [94]. These principles were complemented by focussing on clarity, using concise language and an intuitive layout to minimise cognitive load during high-stress situations. Additional specific design recommendations were derived from the ‘Cognitive Aids in Medicine Assessment Tool’ by Evans, McCahon, Barley, Norris, Khajuria and Moppett 2015 [94]. Goldhaber-Fiebert and Howard 2013 [61] described a four-phase design strategy for emergency tools adopted in this research. Specific emphasis was placed on the involvement of end-users in the design phase using simulation to iteratively test and re-design the tool [95].

### Conceptual design (phase 2)

The initial Team Reflexivity Aid for Cardiac arrest (TRAC) design was based on contemporary resuscitation guidelines [96–98] and consisted of three colour-coded sections: (1) resuscitation summary section, including resuscitation times, patient history and administered therapies, (2) differential diagnosis section and (3) eligibility criteria for extra-corporeal life support (Supplementary material 2).

Review of a multidisciplinary expert panel resulted in the addition of ‘tally marks’ to facilitate documentation of epinephrine and amiodarone administration and the removal of the background colours to enhance contrast and readability.

### Simulation-based testing (phase 3)

**Fig. 2:**
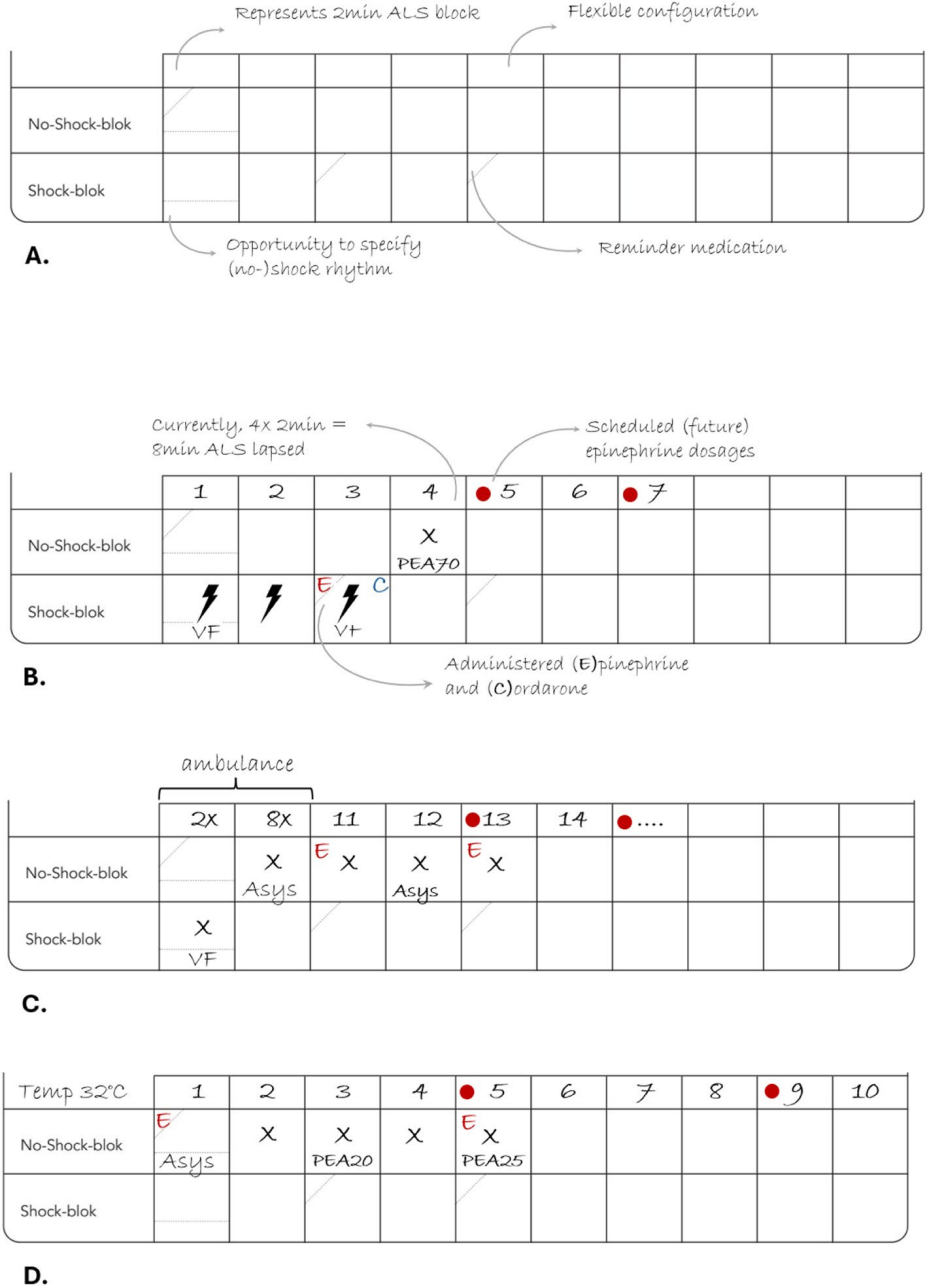
Example of longitudinal resuscitation timelines. Legend: empty longitudinal timeline with cognitive reminders (**A**), example of resuscitation event with administered and anticipated medication (**B**), the opportunity to incorporate prehospital resuscitation blocks (**C**) and anticipation of different epinephrine dosing in hypothermia (**D**)

Three rounds of simulation testing, involving a total of 3 × 10 groups, were conducted from September to December 2023. A significant revision was made to the resuscitation therapy section after users felt that ‘tally marks’ and the general therapy section provided insufficient support. A new longitudinal resuscitation timeline with 2-min blocks was introduced. This timeline initially included hardcoded resuscitation block numbers (e.g. 1st, 2nd, 3rd…) but these were later removed to allow for greater flexibility (see Fig. 2).

Given the variance in local practices reported during simulation testing, an additional section was included to provide a quick review of important local information, such as telephone numbers. Further important lay-out modifications were made to the team reflexivity aid: contrast was enhanced and the patient history and therapy sections were enlarged to better accommodate user handwriting.

**Fig. 3:**
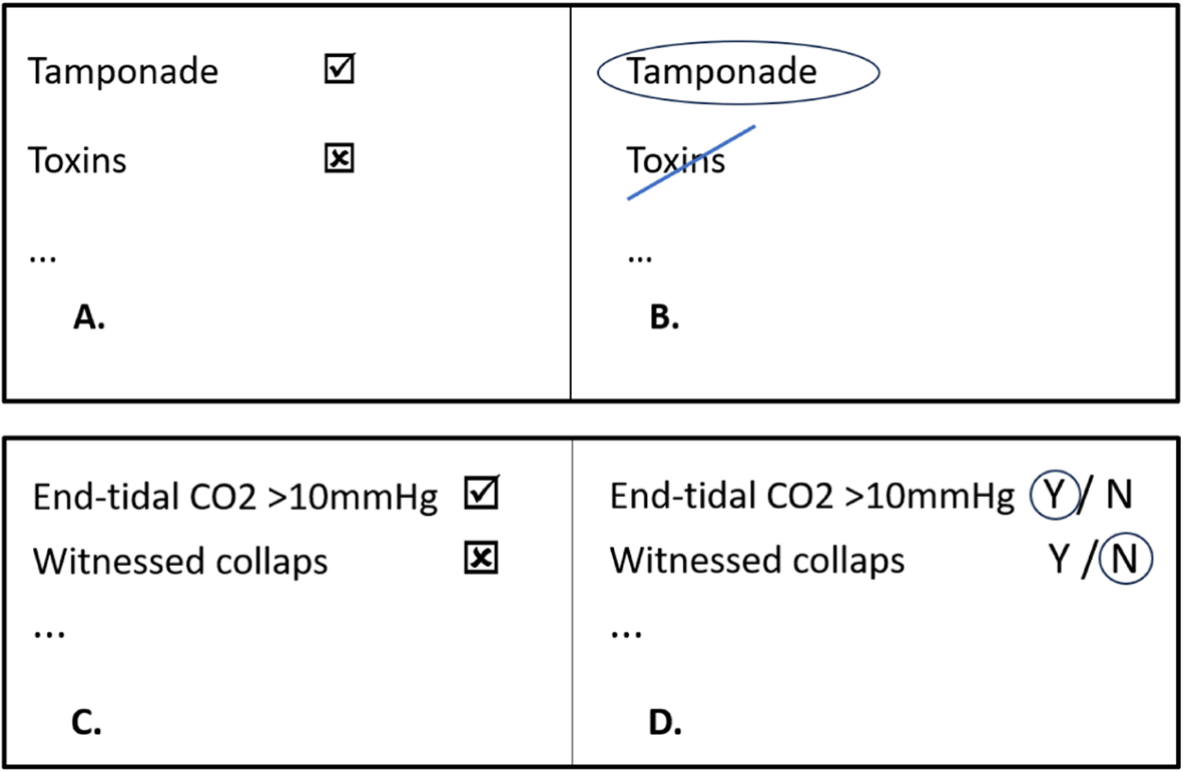
Modifications to facilitate uniform interpretation. Legend: panels **B** and **D** improved ease of uniform interpretation as compared to the corresponding panels **A** and **C**

Pilot testing revealed that using check marks (☑) led to ambiguous interpretations as some users placed check marks whenever an item was evaluated, regardless of its applicability. Additionally, the distinction between ☑ and  ☒ was prone to errors in writing. All checkboxes were therefore replaced with encircling ‘Yes / No’ options and instructions were given to ‘strike-through’ non-applicable items to facilitate correct interpretation (see Fig. 3).

Despite the preference for using generic names for resuscitation drugs, it was found that using (A)drenaline led to conflicting interpretation with (A)miodarone. To avoid confusion, the terms (E)pinephrine and (C)ordarone were advocated.

The team reflexivity aid post-simulation testing consisted of five sections:

1. Relevant resuscitation times and intervals
2. Relevant patient history
3. Resuscitation interventions on a longitudinal timeline
4. Differential diagnosis
5. E-CPR eligibility criteria and important local information.

The final version was made available as an A_1_-sized (85 × 60 cm) reusable whiteboard magnet with a font size range of 8 to 12 mm. An explanatory user guide was written, along with ‘best practice’ examples to facilitate training and implementation.

### Implementation and training (phase 4)

During the year 2023, a total of 60 training sessions were conducted in the hospital, training 300 resuscitation team members, which accounted for 120% of the resuscitation team personnel (i.e. some team members participated more than once).

### Evaluation in practice (phase 5)

#### Survey

The survey was sent to 247 participants, consisting of 87 doctors and 160 nurses. A total of 84 healthcare providers responded (34%). Respondents’ demographics are shown in Table 1. A third (33%) of the respondents were doctors, the majority of whom had a background in intensive care medicine or cardiology. Two-thirds (62%) of the respondents were registered nurses. Most respondents were aged 20–40 years (63%). All resuscitation team roles were represented amongst the respondents. Of all participants, 53 (63%) had experience with the team aid during a real resuscitation. The evaluation of the layout, content and application of the aid is shown in Table 2.

**Table 1:** Participants’ demographics (*N* = 84)

Background	Count (%)
Nurse	52 (62%)
Doctor	28 (33%)
ICU	11 (13%)
Cardiology	8 (10%)
Emergency medicine	4 (5%)
Anaesthesia	2 (2%)
Other	2 (2%)
Age distribution	Count (%)
20–30 years	26 (31%)
30–40 years	27 (32%)
40–50 years	14 (17%)
50–60 years	10 (12%)
60–70 years	5 (6%)
Resuscitation team role	
Airway management (assistance)	17 (20%)
Chest compressions	31 (37%)
Defibrillator and drug preparations	37 (44%)
Overall support	42 (50%)
Registration and documentation	18 (21%)
Team leader	9 (11%)
Consultancy	11 (13%)

**Table 2:** Team aid evaluation

	Mean (SD)*
Layout	
*The team aid is:*	
Properly sized	3.97 (0.70)
Good readable	3.87 (0.78)
Intuitive to use	3.63 (0.93)
Content	
*How useful do you find the content of:*	
Patient history and time section	3.85 (1.03)
Resuscitation event section	4.40 (0.72)
Differential diagnosis section	4.09 (0.79)
ECPR indications section	3.66 (1.04)
Local practice section	3.40 (1.03)
Application	
*The aid results in an improvement of:*	
Communication	3.82 (0.77)
Documentation	4.25 (0.66)
Cognitive load	3.94 (0.68)
Team performance	3.80 (0.76)
Team learning	3.53 (0.73)

^*^5-item Likert scale: strongly disagree (1) to strongly agree (5) for the layout and application questions, not at all useful (1) to very useful (5) for the content questions

Most respondents (*N* = 48, 57%) experienced benefits in communication during the in-action phase, whereas this percentage during the pre- and post-action phases was 26% and 37% respectively. Only a minority (*N* = 22, 26%) had read the accompanying user manual. Of those who had read it, the majority (*N* = 17, 77%) found it (strongly) beneficial.

Two open questions were asked: “What effects does the team aid have on the resuscitation team’s non-technical performance?” and “What effects does the team aid have on the resuscitation team’s technical performance?”. Thematic analysis revealed two overarching themes: ‘improved team performance’ and ‘improved task performance’. The former had three subthemes: ‘improved team situation awareness’, ‘improved communication’ and ‘increased team participation’, each subtheme contained 3 to 5 codes (see Table 3). The latter had two subthemes: ‘improved drug administration’ and ‘improved clinical reasoning’. Each subtheme contained two codes.

**Table 3:** Team aid effect themes

Theme	Subtheme	Codes	Examples [respondent ID, background]
Improved team performance	Improved team situation awareness	Status of case	[Transl.] *“When used properly, it [TRAC] is a very useful structured way to document the course of the resuscitation in a way that keeps everyone informed, especially the circulator and team leader.”* [R31, doctor] [Transl.] *“Provides overview on where case is, what has been done already and what still needs to be done.”* [R2, doctor]
		Status ALS algorithm	[Transl.] *“Clear overview of [ALS] blocks, shock/no shock and medication given.”* [R27, nurse] [Transl.] *“Clear status where you are in CPR [algorithm] and what medication has to be given.”* [R39, nurse]
		Review opportunity	[Transl.] *“[ALS] blocks can be reviewed / read back.”* [R18, nurse] [Transl.] *“Opportunity to review course of the case afterwards.”* [R45, nurse]
		Structure	[Transl.] *“Provides structure in a hectic situation.”* [R28, nurse] [Transl.] *“It provides a nice structure to the [ALS] protocol. In addition, it provides a lot of overview, even with E-CPR requirements.”* [R7, nurse]
		Summarizing	[Transl.] *“Overview for circulator so that brief summaries after rhythm checks are easier to do.”* [R36, nurse] [Transl.] *“Having an overview facilitates summarization”* [R5, doctor]
	Improved communication	Structured communication	[Transl.] *“Communication is more structured.”* [R13, doctor] [Transl.] *“As a circulator, you have a better overview and can therefore better inform the team leader during CPR.”* [R23, nurse]
		Ease of(f) communication	[Transl.] *“More calmness as people can see where the case is, so there is less need to communicate about it.”* [R19, nurse]
		Facilitates consensus	[Transl.] *“[TCAR] contributes to [team] alignment and including everyone in the thought process and achieving consensus in the decision making.”* [R42, doctor] [Transl.] *“Checklist function, did we consider all [differential] diagnoses; are all team members on the same page?”* [R11, doctor]
		Mutual feedback	[Transl.] *“Makes it easier to speak up to each other and remain vigilant as a team.”* [R2, doctor]
		Communication new team members	[Transl.] *“Quick overview for the whole team, easy to catch up on when new colleagues enter the room.”* [R84, doctor]
	Increased team participation	More input	[Transl.] *“The TRAC allows the entire team to oversee the case at a glance, e.g. regarding relevant differential diagnosis, status of the [ALS] block and medication that has been given. As a result, I found that everyone is thinking about the case or preparing for the next step.”* [R24, nurse] [Transl.] *“It [TRAC] contributes immensely to communication, collaborative thinking and situational awareness of the team.”* [R49, nurse]
		Re-engaging	[Transl.] *“It is easier to follow case progression and the current situation. This makes it easier to catch up and contribute again [after being task fixated].”* [R7, nurse]
		Anticipation	[Transl.] *“When used in the shock room [ref. TRAC], the whole team can see where in the [ALS] algorithm we are, this is especially beneficial to the circulation and team leader of course. This allows everyone to anticipate what tasks are expected, what further interventions can be done.”* [R49, nurse]
Improved task performance	Improved drug administration	Verification/reminder	[Transl.] *“Good overview of current [ALS] block with quick overview of which medication is to be administered.”* [R42, doctor] [Transl.] *“Overview of when to administer which medication.”* [R36, nurse]
		Reduced mistakes	[Transl.] *“Less chance of errors through structured documentation of ALS blocks, defibrillations and medication given. Also possible to anticipate next medication administration if the [person documenting] is aware of this option.”* [R31, doctor] *“It [TRAC] is beneficial when transitioning from shock to no-shock blocks and *vice versa*.”* [R48, doctor]
	Improved clinical reasoning	Completeness	[Transl.] *“Checklist function, did we consider all [differential] diagnoses; are all team members on the same page?”* [R11, doctor] [Transl.] *“The [ALS] block overview is nice, also being able to ‘check off’ differential diagnoses helps.”* [R56, doctor]
		In-depth reasoning	[Transl.] *“Clinical reasoning is improved through use of TRAC.”* [R48, doctor] [Transl.] *“Everyone is informed about the case. Everyone is invited to think along and apply broad clinical reasoning.”* [R28, nurse

Important prerequisites of effective team aid use were derived from closing remarks. First, training in the optimal use is important. [Transl.] “I would like lesson(s) on how to optimally use the [team aid] poster.” [R67, nurse] Second, available writing space is limited, especially with prolonged resuscitations and/or whenever a big-tipped marker is used. [Transl.] “There is too little space for filling in the [ALS] blocks. There are only 10 blocks available and with a big-tipped marker is it difficult to write in these.” [R17, nurse] [Transl.] “A smaller marker so you can write on it [TRAC] more clearly.” [R36, nurse] Third, concomitant tasks distract attention from completing the team aid and may lead to documentation errors or omissions. [Transl.] “Often you are writing and then orders are given [by the team leader], so you are interrupted each time and cannot finish your writing (…) This can cause important information to be lost both in the transferred order and the team aid documentation.” [R83, nurse].

Finally, a majority (86%) of respondents expressed a desire to continue using the team reflexivity aid in future resuscitation events.

#### Observations

From January to June 2024, a total of ten resuscitations in the emergency department (i.e. clinical settings) were observed. In all cases, the teams used the team reflexivity aid for documentation and reflection purposes. Critical reflection about differential diagnosis in terms of arrest aetiology was always achieved with help from the team’s aid (10/10). According to the external observers, the team reflexivity aid improved teams’ adherence to the ALS algorithm and supported switching between (no)shock resuscitation blocks in all but one case (9/10).

## Discussion

In this user-centered iterative design study, a Team Reflexivity Aid for Cardiac arrests (TRAC) was developed to facilitate information sharing and in-action reflexivity amongst resuscitation team members. Important aspects were found that we will discuss and link to the literature below as structured per the design phase.

### Phase 1

Of the cognitive aids identified in the literature, only a few were designed to support the entire resuscitation team. As the focus shifts from individual work to healthcare teamwork [99, 100], we see increasing acknowledgment of the importance of coordinating mechanisms and team cognition [12, 25, 101–104]. This shift towards teamwork also requires aids to be evaluated on a team level [17, 59]. Although simulation-based research has many advantages for example in the control of variables and standardisation, important differences with the authentic resuscitation setting require team aids also to be evaluated in clinical practice [10, 83, 105]. Despite this need, only a very limited number of tools have actually been tested in authentic clinical settings.

### Phases 2 and 3

The team reflexivity aid was derived from relevant guidelines identified in the literature [96–98] and modified through iterative testing, as advised for new cognitive aids [34, 61, 85, 86, 93, 94, 106]. The key information sections (e.g. resuscitation times, patients’ status, history and medical interventions) align with previous publications on the resuscitation team’s essential information [7, 107, 108].

Simulation-based approaches play a crucial role not only in the design and implementation of cognitive aids but also in their evaluation to ensure they meet the requirements of end-users [17, 85, 95]. Simulation-based testing and refinement provides an opportunity to collect real-time feedback in a controlled environment, enabling designers to refine and optimise cognitive aids based on user experiences. In our study, simulation-based testing proved invaluable, revealing important design aspects that were modified to enhance functionality and increase acceptability among the target audience. Moreover, it uncovered a crucial additional layer of complexity in team reflexivity designs: the necessity for the aid to simultaneously address the needs of direct (e.g. medical scribes) and indirect users (other team members) [93, 94]. This dual-user consideration has significant implications for design choices, such as avoiding potentially ambiguous elements like checkmarks, and for user training strategies. The effectiveness of a team reflexivity aid is maximised only when annotations are correctly interpreted by all team members. We also applied a specific simulation-based learning methodology, employing a deliberate practice approach, which facilitated user training in time-constrained healthcare settings. This methodology ensured that both direct and indirect users could effectively utilise the cognitive aid in real-world scenarios. This comprehensive approach to design, implementation and evaluation—grounded in simulation—ultimately led to a more robust and user-centred team reflexivity aid.

One might argue that a digital form with direct computer and/or tablet-based input would be more time-efficient since team members could track input on a large video ‘team screen’. We however purposefully chose a ‘paper-like’ version for two reasons. First, we sought to provide users with maximum flexibility for note-taking and annotating [75, 109]. Sarcevic, Zhang, Marsic and Burd 2016 [110] found that over 70% of trauma team leaders made notes on their checklist, with experienced team leaders taking even more notes compared to less experienced colleagues [110]. This externalizing and sharing of information is found most important during complex, knowledge-based processes in order to reduce cognitive load [110]. Second, a ‘paper-like’ version is low-cost and readily available whenever needed in the unpredictable world of emergency medicine and relatively easy to implement in hospitals [71, 111].

### Phases 4 and 5

The extensive initial user training, identical digital form to store data in the hospital’s electronic patient database and integration of the team reflexivity aid in regular training schedules can be regarded as successful implementation since most users had experience with the tool during an actual resuscitation event and were willing to continue its use. However, successful implementation is certainly not self-evident as numbers as low as 10% have been reported with paper-based aids before [83, 112]. Our implementation process underscores two known aspects: (a) user manuals, despite being referenced in training, were seldom consulted and cannot be relied upon for effective implementation [83] and (b) comprehensive user evaluation and feedback are essential to bridge the gap between the intended (“as designed”) and actual (“as done”) application of a new team tool [113, 114]. When the TRAC became more versatile over time, a growing emphasis was placed on user training to ensure that application “as done” remained aligned with application “as designed”. This shift highlights a delicate balance between intuitive simplicity and multiplicity requiring more training. Perceived usability was highest for the resuscitation event and differential diagnosis sections. This could be explained by the fact that these sections require most information sharing and reflexivity amongst team members, in comparison to the E-CPR eligibility and local information sections which are more ‘static’ reminder sections. Additionally, the hospital’s E-CPR eligibility criteria differed slightly from those derived from the literature, underscoring Fletcher and Bedwell’s [34] recommendation that cognitive aids need full adaptation to local operating procedures.

We found that a dedicated group of direct users that can be extensively trained was preferable for two reasons. First, training enhances interpretability by the rest of the team which has to do with structuring notes and using unambiguous text. Second, distractions and/or concomitant tasks of the direct user (i.e. medical scribe) may result in note-taking omissions or delays that can have negative implications for team and/or task performance [115].

The perceived improvement in teams’ situation awareness found in this study extends the previous work on team aids and situation awareness [9, 75, 116]. Situation awareness is known to be influenced by the context in which the aids is evaluated, with higher task loads or disruptive team collaboration contexts generally leading to more perceived benefits, which could explain some positive effects found in our evaluation that were absent in the study by Parush, Mastoras, Bhandari, Momtahan, Day, Weitzman, et al. 2017 [9]. Furthermore, team situation awareness is complex as it not only involves the perception, comprehension and projection of the team’s situation at hand but also relates to other team aspects such as inclusive leadership, good followership and communication principles (e.g. closed loop communication, speaking up) [75, 117].

The perceived improvement in communication aligns with the major effect reported by Crabb, Hurwitz, Reed, Smith, Martin, Tyndall, et al. 2021 [69] with their electronic clinical decision display system: displayed information does not need to be verbalised, thereby reducing the cognitive burden of the team leader, allowing more focus on aspects such as task performance or team coordination [9, 118]. This applies to information sharing within the team, but explicitly also to new members entering the team [71]. Kolbe 2009 [101] analysed team coordination patterns and defined explicit and implicit coordination, with the former being intentionally used for the purpose of team coordination either as verbal or written communication. Implicit coordination is based upon shared cognition and anticipation of future actions by individual team members and requires both a shared and accurate mental model [101]. The TRAC tool can enhance team communication providing a shared ‘cognitive framework’ for the resuscitation event. By displaying the resuscitation chronology, administered therapies and relevant working diagnoses, the tool enables team members to structure their communication more effectively, pose clarifying questions and contribute through co-construction and constructive conflicts [32]. This functionality is particularly beneficial for team members who may have been engaged in high-demand tasks, preventing their participation in time-delineated reflexivity processes such as the’10 sec for 10 min’ team huddle [119]. Consequently, the TRAC tool not only facilitates team reflexivity by providing team members with a better understanding of the situation but also extends reflexivity opportunities by providing individuals with continuous access to key information. Furthermore, users are encouraged to not see the team reflexivity aid as a unidirectional information page, but rather as a prompt and blackboard for discussion [117]. This applies especially to the differential diagnoses section. Verbalisation of these aetiologies might be easier as the items are listed on the tool; however, the structure can also provide other members information on *what to expect* and time to *think ahead* which might benefit team input, especially under time constraints [17].

The reported benefit of the TRAC tool on taskwork, despite its lack of explicit decision support, is noteworthy and aligns with findings from other team aids that similarly lack explicit treatment directions [9, 75]. Calder, Bhandari, Mastoras, Day, Momtahan, Falconer, et al. 2018 [75] observed that the presence of a team screen significantly reduced communication, particularly when new members joined the team, thereby minimizing distractions for the team leader and allowing greater focus on team and task performance. In a related study, Parush, Mastoras, Bhandari, Momtahan, Day, Weitzman, et al. 2017 [9] found that while the overall amount of communication exchanges remained similar with and without a team screen, its presence led to a reduction in communication episodes regarding patient status and an increase in discussions about interventions. This intertwining of taskwork and teamwork could explain why, despite the TRAC’s clear taskwork focus, its perceived effects extended to team performance. The relationship between information sharing (i.e. team reflexivity), creating an accurate shared mental model and subsequent team performance is well-established in the literature [25, 31, 100, 103, 120]. This study extends our understanding of this concept by highlighting that even a taskwork-focused team aid may benefit teamwork. A similar effect was observed by Marshall and Mehra 2014 [121] who found improved team performance with a displayed linear ‘cannot intubate, cannot oxygenate’ task-focused cognitive aid.

Possible explanations for this phenomenon relate to the observation that task-focused reflexivity enhances situation awareness by increasing the team’s understanding of the current situation as well as tasks and goals at hand [121], thereby aligning the team with necessary immediate actions. A taskwork-oriented reflexivity tool can also reduce mental workload and/or reduce the need for communication, leaving more mental capacity available for monitoring and coordinating team performance [121]. Finally, a task-focused reflexivity tool can enhance collaboration by providing team members with a more universal shared external framework that supports coordinated collective action. The team FIRST framework also summarises communication skills and team reflection as enablers for optimised team mental models with subsequently enhance team performance [100]. While the TRAC tool can facilitate information sharing within resuscitation teams, it is crucial to recognise underlying concepts for effective team communication and psychological safety are prerequisites [122]. Research has shown that inclusive leadership, open culture, and flattened hierarchies are key facilitators for information sharing in healthcare teams [123–127].

## Strengths, limitations and future research

### Strengths

To our knowledge, this is the first team reflexivity aid specifically designed for team information sharing during cardiac arrests. The study shows the encouraging effects of a low-cost, easily available team aid with perceived benefits to the task and teamwork.

### Limitations

The research on the team reflexivity aid presents important limitations that affect its possible applicability. These include the following:

Team size and manpower dependency: larger teams, as in our study, generally benefit more from team reflexivity and have more manpower available to handle additional task loads as required for team aids [31, 46]. This limits generalisability to smaller teams.

Expertise requirement: the lack of decision support with this reflexivity aid, requires knowledgeable and experienced resuscitation teams.

Visibility constraints: the team reflexivity aid requires all team members to have visual access, which requires a predefined set-up and limits applicability to resuscitations in other settings, such as prehospital. Smaller versions were made available for resuscitation code carts (A_4_-sized) and as personal pocket version (A_6_-sized), however as other team members cannot (easily) see these sizes, the function alters from a team reflexivity aid to a personal documentation aid.

We preferably observed only real-life patient resuscitation events in the emergency department; although, during the study period, only a few out-of-hospital cardiac arrests occurred within working hours. We therefore added some of the hospitals’ in-situ process evaluations where simulated cardiac arrest patients are presented in the authentic emergency department context for evaluation purposes. These in-situ evaluations are more representative of the authentic context compared to off-site simulation-based evaluations; however, the training concept might still alter perceived stress as compared with real patients and relatives [10].

### Future research

This research shows promising effects of a team reflexivity aid, although more research is required. Research exploring the concept of how reflexivity aids can initiate, structure, and/or improve team reflexivity is needed in order to understand the theoretical concept and its possible effects on task- and teamwork better as well as its modifiers. This includes the identification of relevant variables such as necessary team size, time constraints, team members’ experience, task complexity and/or context as well as team communication aspects such as psychological safety and speaking up.

## Conclusion

This study demonstrates the successful development and implementation of the Team Reflexivity Aid for Cardiac arrests using simulation methodology. This task-focussed whole team tool was found to improve perceived team situation awareness, communication, and overall performance. The research highlights the intricate interplay between task- and teamwork in healthcare settings, underscoring the potential for taskwork-oriented tools to yield significant benefits for team dynamics. These findings warrant further investigation into team-supportive interventions and their impact on resuscitation outcomes.

## Supplementary Information

Supplementary material 1. Overview cognitive aids used during cardiopulmonary resuscitation [128–176]

Supplementary material 2. Iterative TRAC design over time

## Data Availability

No datasets were generated or analysed during the current study.
