# Supplementary material for: Getting ON-TRAC, a team-centred design study of a reflexivity aid to support resuscitation teams’ information sharing
Source: Adv Simul (Lond). 2025 Mar 28;10:17. doi: 10.1186/s41077-025-00340-8 (PMC11951662; doi:10.1186/s41077-025-00340-8)
Supplement: Supplementary file 1 — Supplementary material 1. Overview cognitive aids used during cardiopulmonary resuscitation [128–176] [file 41077_2025_340_MOESM1_ESM.docx]

**Online supplement 1, Overview cognitive aids used during cardiopulmonary resuscitation**

| **First author (year)** | **Cognitive aid** | **Cognitive aid description** | **Resuscitation level** | **Designed for** | **Study setting** | **Participants** | **Outcome measure(s)** |
| --- | --- | --- | --- | --- | --- | --- | --- |
| **Memory aids – mnemonics, figures, …** | |  |  |  |  |  |  |
| Fijacko 2024(128) | Five-finger mnemonic | 5-finger mnemonic for teaching the theoretical aspects of adult BLS steps | BLS | Individual HCP | Simulation | 13 schoolchildren | Theoretical knowledge on adult BLS |
| Coggins 2018(129) | C.O.A.C.H.E.D. defibrillation | Acronym describing the sequential order of steps during emergency defibrillation | ALS | Individual HCP | Simulation | 66 registered nurses, 25 senior doctors and 21 registrars | Length of peri-shock pause and safety of defibrillation practice |
| Dyson 2004(130) | EMD-aide figure | Common EMD causes organized in 4 groups by frequency of occurrence and ease of reversibility | ALS | Individual HCP | Telephone interviews | 119 house officers | The number and sequence of recall within one minute and overall |
| **Team member role allocation** | | |  |  |  |  |  |
| Chong 2024(72) | A-C-L-S teamwork model | Person, task, tool, position and mnemonic allocation per resuscitation team member | ALS | Resus team | Emergency department | 104 cardiac arrests | Implementation through simulation training Video-recorded ED resuscitations analysed for team composition, timing of tasks and chest compression fraction |
| Kim 2021(76) | Numbered jerseys | Comparison between regular work attire compared to numbered jerseys | ALS | Resus team | Simulation | 48 internal medicine residents divided in 8 teams of 6p | Amount of directed commands  Teamwork scores and time to critical actions |
| Di Renna 2016(73)  Di Renna 2013(70) | Cognitive Aids with Roles Defined (CARD) | Concept focusing on clarification of individual roles and distribution of tasks through individual cards worn by team members | A(P)LS | Intraoperative resus team | Simulation | 24 teams-consisting of 12 HCPs | Design using simulation and focus group interviews Follow-up simulation analysis after 6 months on team and CPR performance (TEAM score, time to initiation CPR and hands-off time) |
| **CPR Feedback support** | | |  |  |  |  |  |
| Stumpf 2022(131) | Smartphone app | Feedback on chest compression derived from smartphone’s intrinsic accelerometer together with auditory and haptic feedback | BLS | Individual HCP | Simulation | 60 sessions, not otherwise specified | Chest compression rate and depth |
| Sevil 2021(132) | PocketCPR app by Zoll® | Smartphone’s accelerometer derived feedback on chest compression depth and rate with visual and aural feedback | BLS | Individual lay rescuer | Simulation | 137 first-year students | Technical chest compressions performance (rate, compression, depth, recoil) |
| Plata 2019(133) |  |  |  |  | Simulation | 100 lay rescuers | Technical chest compressions performance (onset, depth, rate, interruptions, hand positioning, recoil) |
| Eaton 2018(134) |  |  |  |  | Simulation | 118 lay rescuers | Technical chest compressions performance (onset, depth, rate, interruptions, hand positioning, recoil) and ventilation attempts and rate |
| Park 2014(135) |  |  |  |  | Simulation | 64 lay rescuers | Chest compression accuracy (e.g. depth, recoil, hand positioning) and questionnaire on accuracy awareness on chest compression |
| Dong 2020(136) | TCPRLink® Laerdal  (also referred to as  QCPR cam app) | Using smartphone’s front camera, the app detects chest compressions and calculates hands-off time, compression rate, displaying this to the lay rescuer and transmitting it over the internet to EMS dispatchers | BLS | Individual lay rescuer | Simulation | 186 lay rescuers | Technical performance of chest compressions (elapsed time, rate, depth, interruptions, recoil) |
| Meinich-Bache 2018(137) |  |  |  |  | Simulation | 19 technical evaluations | Performance in five categories: smartphone position, outdoor environment, disturbances, random movements and summary reports |
| Meinich-Bache 2017(138) |  |  |  |  | Simulation | 7 lay rescuers performing 15 technical evaluations | Performance in three categories: noise due to hair, noise due to bystander and noise due to random movements |
| Brown 2018(47) | CPR Card or CPR-quality VisF device (Laerdal Corp) | Credit card sized CPR visual feedback device, using accelerometer technology to provide LED feedback on depth and rate of chest compressions | APLS | Individual HCP | Simulation | 108 teams of 3p (324 HCPs) | Workload (NASA TLX), CPR performance data |
| Cheng 2015(139) |  |  |  |  | Simulation | 108 teams of 3p (324 HCPs) | CPR performance data (chest compression depth, fraction and rate) |
| Cheng 2018(140) | CPR Coaching | Using CPR feedback defibrillator (with/without CPR coach), | APLS | Individual HCP | Simulation | 200 HCPs from paediatrics or emergency care divided in 5p teams | CPR performance indicators (delays, chest compression fraction, depth, durations) |
| **Decision support - applications on hand-held devices (smartphone, tablet, …)** | | |  |  |  |  |  |
| Rückert 2025(141) | eGENA app | electronic cognitive aid for emergencies in anaesthesia | ALS | Individual HCP | Simulation | 9 teams of 4p | Resuscitation performance scores, cooperation doctors – nurses, self-assessed safety |
| Schild 2019(142) |  |  |  |  | Simulation | anaesthesiology staff (specialists & residents), total of 29 | User centred design with evaluation of prototypes through System Usability Scale, think-aloud analysis and user questionnaire |
| Benguigui 2024(143) | Smartphone application Medical Assistance eXpert (MAX1 and MAX2) | App designed as linear and sequential cognitive app containing step-by-step guidance with visual and vocal instructions | NLS | Individual HCP | Simulation | 108 residents (from paediatrics and midwifery) divided in 3p teams | Technical performance (NRP checklist) and nontechnical skills (TEAM score) and participants perception (questionnaire) |
| Donze 2019(43) |  |  | ALS | Individual HCP | Simulation | Anaesthesia residents | Technical performance on 13-18 critical actions per scenario and Non-technical performance on Ottawa Global Rating Scale |
| Lelaidier 2017(44) |  |  |  |  | Simulation | 46 anaesthesia residents |  |
| Cheng 2024(74) (study protocol) | InterFACE tool | As below, extended with role-specific decision-support for the team leader and medication nurse wearing AR devices | APLS | Resus team | Simulation | 80 teams of consisting of 5 HCPs | Delay in critical interventions, CPR performance indicators, team performance (TEAM), workload (NASA Task-Load Index), cognitive load (PAAS), user experience, system usability, provider anxiety, technology acceptance |
| Ehrler 2021(68) |  | Interconnected and Focussed Mobile device Application in the Patient Care Environment: ‘Guiding Pad’ interface to remote large team LCD screen |  |  | Simulation | 48 HCPs divided into 6p-teams | Situational awareness (SAGAT), leadership (RTLE), team performance (TEAM), delays in action and medication errors, participants’ perceived usability |
| Ehrler 2018(77) |  |  |  |  | Mixed | Focus groups Field observations Interviews | Distillation design requirements and  prototype development |
| Senter-Zapata 2024(144) | ACLS app | Guided ACLS app | ALS | Individual HCP | Simulation | 46 residents | CPR success (ROSC), guideline deviations, experienced stress and user confidence |
| Corazza 2023(145) | PediAppRREST app | Interactive, multimodal (audio-visual), checklist app, sequentially displaying prompts on recommended APLS interventions | APLS | Individual HCP | Simulation | 300 residents divided over 100 teams | Number of deviations from APLS guideline (delays and errors), CPR quality, System Usability Scale,  Workload (NASA Task Load Index) and team performance (Clinical Performance Tool) |
| Corazza 2021(146) |  |  |  |  | Simulation | 315 residents divided over 105 teams | Number of deviations from APLS guideline (delays and errors), CPR quality, System Usability Scale,  Workload (NASA Task Load Index) and team performance (Clinical Performance Tool) |
| Corazza 2020(147) |  |  |  |  | Simulation | 48 paediatric residents divided over 16 teams | User Experience Questionnaire, qualitative feedback, APLS guideline adherence, workload (NASA Raw-Task Load Index) and team performance (Clinical Performance Tool) |
| Watkins 2022(81) | Comparison of three different cardiac arrest decision-support tools (DST) | Three DST on iPad mini were compared:   - Technical DST - Non-technical DST - Combined DST   According to APLS guideline | APLS | Individual HCP | Simulation | 35 teams of 4-5p | Technical performance (assessment tool) and  Non-technical performance (TEAM and BARS) |
| Grundgeiger 2021(42) | Cognitive Aid app (CaApp) | Updated version of ‘DoRea’ app to better suite the experienced users with time keeping, reminders and documentation features. | ALS | Individual HCP | Simulation | 67 experienced 2p-teams (emergency physician and qualified nurse) | Aggregated CPR performance score and  NASA TLX raw |
| Grundgeiger 2019(59) |  |  |  |  | Simulation | 3p resuscitation teams of anaesthesiologist and 2 team members | Aggregated technical performance score (9-10 items per scenario) and  Team Emergency Assessment Measure |
| Grundgeiger 2016(108) | DoRea app | Tablet-based real-time documentation tool for in-hospital resuscitations | ALS | Individual HCP | Simulation | 26 staff anaesthesiologists | Precision, completeness and amount of time for resuscitation documentation |
| Reinhardt 2015(107) |  |  |  |  | Mixed | Resuscitation teams senior anaesthesiologists | Observation of resuscitation teams in simulation,  semi-structured interviews and  pluralistic walk-throughs |
| Lacour 2021(148) | Pediatric Accurate Medication in Emergency Situations (PedAMINES) app | Multiple resuscitation drugs, doses automatically adapted to weight or age entered by user | APLS | Individual HCP | Simulation | 150 advanced paramedics | Participant’s perceived stress |
| Siebert 2021(149) |  |  |  |  | Simulation | 150 advanced paramedics | Rate of medication error and Time to drug preparation and delivery |
| Siebert 2019(150) |  |  |  |  | Simulation | 128 nurses | Medication error proportions |
| Siebert 2017(151) |  |  |  |  | Simulation | 20 nurses | Time to drug preparation and delivery Medication errors |
| Mahajan 2021(152) | AAAIM application | Ambient, augmented, artificial intelligence in medicine application showing decision support steps and safety checks (e.g. drug doses) | ALS | Individual HCP | N/A | N/A | N/A |
| Metelmann 2021(49) | HELP Notfall app | Smartphone based app with step-by-step guidance with visual and acoustic instructions | BLS | Laypersons | Simulation | 200 secondary school pupils | Aggregated technical performance score (amongst others chest compression rate and depth, hand and body position) |
| Müller 2021(153) | CprPrototype app | Algorithms for shockable and non-shockable rhythms with appropriate action support (e.g. drug calculation and preparation) and automatic time keeping and documentation | ALS | Individual HCP | Mixed | Interviews (N=5 HCP), observations (N=15 HCP) and questionnaire (N=83 HCP) | Defining one-dimensional and attractive CPR app attributes according to Kano model. Also HCP’s satisfaction with CprPrototype app |
| Roitsch 2021(46) | NeoCue® (MedicalCue, Inc, Mountain View, CA) | Tablet-based system, uses patient’s heart and respiratory rate to provide auditory and visual prompts with specific interventions according to NLS algorithm. | NLS | Individual HCP | Simulation | 109 HCPs divided into 2 vs 3p-teams | HCP’s cognitive load measured through the National Aeronautics and Space Administration Task Load Index |
| Roitsch 2020(40) |  |  |  |  | Simulation | 109 HCPs divided into 2 vs 3p-teams | Adherence to NLS guideline through modified Neonatal Resuscitation Performance Evaluation |
| Fuerch 2015(154) |  |  |  |  | Simulation | 65 health care providers | Adherence to NLS guideline (e.g. initiation and cessation of positive pressure ventilation and chest compressions and FiO2 adjustments) |
| Cavallin 2020(155) | NeoTapAS | Application designed for registration of neonatal resuscitation with HR calculation according to screen tapping feature | NLS | Individual HCP | Simulation | 20 teams of 3 paediatric residents | Timing of HR establishment and critical interventions (e.g. positive pressure ventilation, chest compressions, intubation, epinephrine administration) |
| Hejjaji 2020(53) | Redivus Code Blue app (Redivus Health Inc) | Activation of the app initiates 2 sets of timers: one for 2-min CPR cycles prompting pulse checks, the other for defibrillation or vasoactive medication. Additionally, guidance on interventions or drug dosing is available. | ALS | Individual HCP | Simulation | 53 ALS-certified internal medicine house officers | Overall compression fraction and number of (in)correct ALS interventions |
| Márquez-Hernández 2020(156) | AsistenteCPR app | Smartphone based descriptive guide to the steps recommended to correctly perform BLS through visual, spoken, and written messages | BLS | Individual lay rescuer | Simulation | 128 students | BLS guideline adherence according to 10-item observation checklist and technical CPR performance measures |
| Siebert 2020(157) | Guiding Pad app | Interactive APLS algorithm with detailed information and cognitive aids to help decision making, calculate appropriate energy levels and drug amounts as well as documentation of interventions | APLS | Individual HCP | Simulation | 26 paediatric residents | Delays and errors in critical interventions (CPR, defibrillation, drugs) |
| Chan 2019(158) | NRP Prompt app | Mobile application that provides audiovisual prompts based on user responses at decision points in the NRP algorithm, | NLS | Individual HCP | Simulation | 39 junior residents dived in 2p-teams | NLS technical performance according to checklist score and elapsed time to critical interventions |
| Jones 2019(52) | TeamScreen electronic Decision Support System (eDSS) | Hand-held tablet coupled with large screen for whole team displaying ALS support process and prompts when treatment is (over)due | ALS | Resus team | Simulation | 34 nurses divided over 8 teams | Protocol adherence Participant’s performance perception |
| Siebert 2017(159) | Augmented Reality Glasses versus APLS pocket reference card | APLS algorithm divided in small glass compatible information ‘cards’ navigating along the original algorithm’s using tactile commands | APLS | Individual HCP | Simulation | 20 paediatric residents | Delays and errors in critical interventions (CPR, defibrillation, drugs) |
| Sakai 2015(160) | CPR support application | Application designed for general public with animation explaining EMS activation, CPR hand positioning, compression depth / rate according to metronome-like sound | BLS | Individual lay rescuer | Simulation | 84 lay rescuers | Proportion of appropriate chest compressions |
| Field 2014(161) | Electronic Decision Support Tool for ACLS | App with programmed sequential management steps according to current ALS guideline | ALS | Individual HCP | Simulation | 47 ACLS-certified senior medical students | Performance of critical actions through errors of omission and commission |
| Low 2011(56) | iResus application by Resuscitation Council UK | App containing adult and paediatric algorithms in an interactive format | ALS | Individual HCP | Simulation | 31 ALS-trained doctors | Overall cardiac arrest performance test score (24 items) |
| **Decision support & patient status overviews on large screens** | | |  |  |  |  |  |
| Crabb 2021(69) | ACLS Clinical Decision Display System (CDDS) | Interactive web-based application with large screen for whole team to view.  CDDS displays and records time intervals for e.g. rhythm checks and epinephrine administration. CDDS documents all interventions and provides summary at the end | ALS | Resus team | Simulation | 56 HCPs divided over 8 teams | Time deviations from guideline recommended actions (rhythm check, defibrillation, drug administration)  Participant’s perception of team organization and communication |
| Shear 2019(50) | Dynamic electronic cognitive aid with clinical decision support versus static cognitive aid | Large screen-based interactive cognitive aid with embedded emergency algorithms versus laminated emergency cards (static) | A(P)LS | Individual HCP | Simulation | 34 anaesthesia residents | Task checklist performance and Anaesthesia Non-technical skills score |
| Calder 2018(75) | Situational Awareness Display | Display with central patient figure on which icons can be placed (as intervention documentation), coupled with patient demographics and clinical history, vital signs, timeline and assigned team members | ALS | Resus team | Mixed | Focus groups  (20 HCPs) and simulation testing (13 HCPs) | Design and evaluation (user perception) of team display |
| Parush 2017(9) | Emergency department resuscitation situation display | Large screen displaying six high-priority information categories (patient history, current status, team members, assistance, interventions and timeline) | ALS | Resus team | Simulation | 13 HCPs divided over 3 teams | Team performance (Clinical Teamwork Scale), situation awareness and team communication |
| Gonzales 2016(71) | Visual TASK | Collaborative cognitive aid presenting relevant tasks on a shared display for teams | APLS | Resus team | Simulation | 23 HCPs  (including nurses, physicians and respiratory therapists) | Interaction with the system (through attentional foci analyses, observation and survey), |
| Hunt 2013(162) | Voice Activated Decision Support System (VADSS) | Computer application that requires user’s verbal response to specific prompts, thereby generating audio and video feedback to promote initiation and performance of CPR | BLS | Individual lay rescuer | Simulation | 31 lay rescuers | Technical performance: amongst others the correctness and delays in chest compression, head-tilt and ventilation and relieve of CPR rescuer |
| Schneider 1995(163) | “Helper” software | computer-based system to exhibit precompiled response plans for medical emergencies with user input through touch screen navigation | ALS | Individual HCP | Simulation | 39 anaesthesia residents | Technical performance (correctness, speed and amount) of ALS interventions (e.g. defibrillation, drug administration, chest compressions) |
| **Decision support - paper-based (reference cards, handbooks, checklists, …)** | | |  |  |  |  |  |
| Ghazali 2023(164) | CPR Checklist | Paper-based checklist with 5 components high-quality CPR and 2-rescuers arrest algorithm | APLS | Individual HCP | Simulation | 48 HCPs divided into 2p-teams | CPR global performance, respiratory and circulatory scores displayed by Laerdal® QCPR™ |
| Goldhaber-Fiebert 2023(82) | Stanford Emergency Manual for perioperative critical events | Handbook with management steps on 26 different life-threatening events | ALS | Individual HCP | Survey | Anaesthesia teams | Emergency manual use over 6-year period |
| Goldhaber-Fiebert 2016(35) |  |  |  |  | Survey | Anaesthesia residents | Self-reported successful use of emergency manual in the OR |
| Urman 2021(165) |  |  |  |  | Simulation | 304 anaesthesiologists | Behaviourally anchored rating scale on six evaluation categories, with (non-)technical performance steps and EM usage |
| Passalacqua 2023(78) | Drug poster | Indications, doses, infusion starting rates, and cautions for resuscitation drugs | ALS | Resus team | N/A | N/A | N/A |
| Jacoby 2022(166) | APLS pocket card | PALS pocket card analysis | APLS | Individual HCP | Mixed | 16 HCPs | Simulation based video analysis with eye-tracking data and semi-structured interviews on card use |
| Shah 2021(167) | Code Team Leader Card | A double-sided 12x18” laminated code card for the team leader to help team leader identification and provide PALS algorithm | APLS | Individual HCP | Simulation | 131 paediatric residents, divided in teams of 5-6p | Code team leader recognition, simulation and teamwork experience (survey) |
| Brune 2020(168) | Cognitive aid card for epinephrine preparation | Visual instructions to syringe assemblage and weight-based table with doses | APLS | Individual HCP | Simulation | 100 nurses | Epinephrine preparation time and error rate |
| Hall 2020(37) | Trial of Emergency Medicine Protocols in Simulation Training (TEMPIST) | Handbook of step-by-step pathways designed to be read out loud during a resuscitation event. | A(P)LS, NLS | Individual HCP | Simulation | 75 HCPs divided into 21 teams | Error rates of key tasks per scenario Participants’ experiences |
| Koers 2020(169) | Cognitive Aids for the Management of Deteriorating Surgical patients (CAMDS) | 16 symptom-specific cognitive aids and 6 general algorithms in A4 size handbook | ALS | Individual HCP | Simulation | 25 teams of 3p (surgeon and 2 nurses) | Omission of key management steps Participants’ perceived usability |
| Rifai 2020(170) | Comparing three graphical CPR protocol cards | ILCOR guideline, Cardio cerebral resuscitation (CCR) protocol and Arnsberg algorithm | ALS | Individual HCP | Simulation | 940 residents, divided in 4p teams | Technical CPR performance data |
| Nelson McMillan 2018(54) | Comparing two different CPR cards containing different algorithms | Comparison AHA PALS Aid card  (4 algorithms different pages) versus Johns Hopskins Kids Kard aid  (3 algorithms single page) | APLS | Individual HCPs | Simulation | 132 paediatric residents | Initiation of CPR and CPR performance (critical interventions) |
| Nelson 2008(51) |  |  |  |  | Simulation | 60 paediatric residents | Task performance (errors in management) |
| Kottmann 2017(171) | Avalanche Victim Resuscitation Checklist | Decision flow-chart for initiation of CPR in avalanche victims | BLS | Individual HCP | N/A | N/A | N/A |
| Kottmann 2015(172) |  |  |  |  | N/A | N/A | N/A |
| Arriaga 2013(18) | Operating room crisis checklist packet | Booklet of 10 specific and 2 general crises | ALS | Individual HCP | Simulation | 17 OR teams with anaesthesia and surgical staff and OR nurses). | Adherence to critical care processes  Participants’ perceptions usefulness |
| Ziewacz 2011(173) |  |  |  |  | Simulation | 2 OR teams, 11 HCPs | Omissions of key management steps Participants’ perception |
| Bould 2009(60) | Neonatal resuscitation guideline poster | Algorithm of neonatal life support protocol | NLS | Individual HCP | Simulation | 32 anaesthesia residents | Correctly performed life-saving interventions |
| Mills 2004(83) | Modified American Heart Association’s Handbook of Emergency Cardiovascular Care | Tabbed handbook for resuscitation code cart | ALS | Individual HCP | Survey | 565 HCPs | Dissemination and participants’ accepted usefulness of cognitive aid |
| Ward 1997(174) | Two checklists (pocket card and extensive handbook version) | Guidance for correct CPR steps | BLS | Individual lay rescuer | Simulation | 169 undergraduates | Overall performance as well as procedural and compression–ventilation key performance steps |
| **Decision support - audio prompts** | | |  |  |  |  |  |
| Dinur 2021(175) | Audio-voice guidance application | Application asks resuscitation questions. According to user’s verbal input, another question or recommended action is provided | NLS | Individual HCP | Simulation | 20 team leaders (10 nurses, 10 residents) | Guideline adherence and elapsed time |
| Merchant 2010(176) | Cell phone prompts | Prerecorded audio prompts as cardiopulmonary resuscitation instructions (uploaded as voicemail) | BLS | Individual lay rescuer | Simulation | 160 participants | CPR quality metrics (pauses, compression rate, depth and hand placement) |

Abbreviations: (P)BLS = (Paediatric) Basic Life Support, A(P)LS = Advanced (Paediatric) Life Support, ACLS = Advanced Cardiac Life Support, NLS = Neonatal Life Support, (N)TP = (non-)technical performance, HCP = Health Care Provider, WP = workplace, SAGAT = situation awareness global assessment technique, RTLE = resuscitation team leader evaluation, TEAM = team emergency assessment measure, CPR = cardiopulmonary resuscitation, EMS = emergency medical service
