# Supplementary material for: Getting ON-TRAC, a team-centred design study of a reflexivity aid to support resuscitation teams’ information sharing
Source: Adv Simul (Lond). 2025 Mar 28;10:17. doi: 10.1186/s41077-025-00340-8 (PMC11951662; doi:10.1186/s41077-025-00340-8)
Supplement: Supplementary file 2 — Supplementary material 2. Iterative TRAC design over time [file 41077_2025_340_MOESM2_ESM.docx]

**Online supplement 2, Iterative TRAC design over time**

| 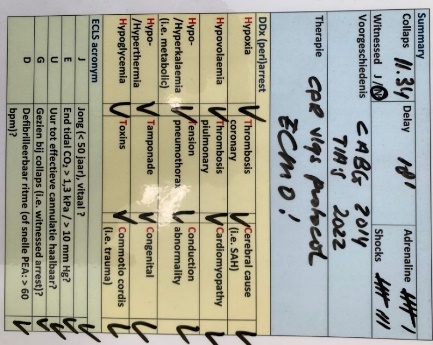 **A** | 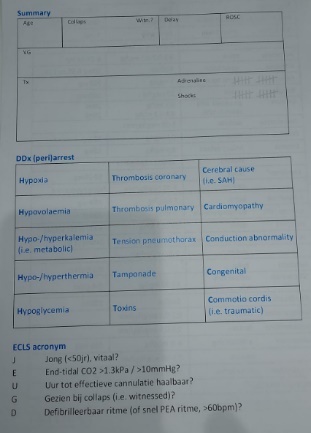 **B** |
| --- | --- |
| 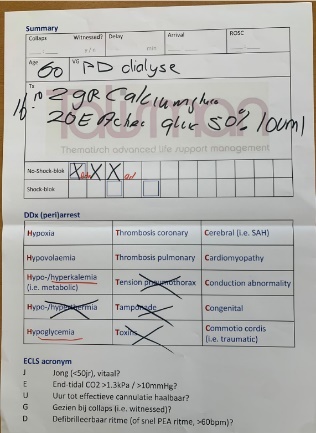 **C** | 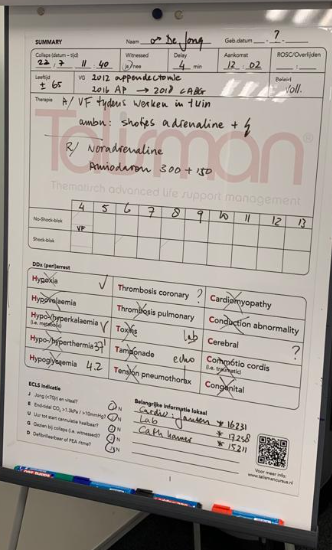 **D** |

**Legenda**: Iterative modifications over time: (a) initial design in phase 2, (b-c-d) iterative modifications resulting from phase 3
